# A Chinese Face Dataset with Dynamic Expressions and Diverse Ages Synthesized by Deep Learning

**DOI:** 10.1038/s41597-023-02701-2

**Published:** 2023-12-07

**Authors:** Shangfeng Han, Yanliang Guo, Xinyi Zhou, Junlong Huang, Linlin Shen, Yuejia Luo

**Affiliations:** 1 School of psychology, Magnetic Resonance Imaging Center, China-UK Visual Information Processing Laboratory, Institute of Computer Vision, College of Computer Science and Software Engineering, Shenzhen University, Shenzhen, China; 2 Department of Psychology and Center for Brain and Cognitive Sciences, School of Education, Guangzhou University, Guangzhou, China; 3 State Key Laboratory of Cognitive Neuroscience and Learning, Beijing Normal University, Beijing, China; 4 School of Psychology, Sichuan Center of Applied Psychology, Chengdu Medical College, Chengdu, China; 5 Institute for Neuropsychological Rehabilitation, University of Health and Rehabilitation Sciences, Qingdao, China

**Keywords:** Psychology, Social sciences

## Abstract

Facial stimuli have gained increasing popularity in research. However, the existing Chinese facial datasets primarily consist of static facial expressions and lack variations in terms of facial aging. Additionally, these datasets are limited to stimuli from a small number of individuals, in that it is difficult and time-consuming to recruit a diverse range of volunteers across different age groups to capture their facial expressions. In this paper, a deep-learning based face editing approach, StyleGAN, is used to synthesize a Chinese face dataset, namely SZU-EmoDage, where faces with different expressions and ages are synthesized. Leverage on the interpolations of latent vectors, continuously dynamic expressions with different intensities, are also available. Participants assessed emotional categories and dimensions (valence, arousal and dominance) of the synthesized faces. The results show that the face database has good reliability and validity, and can be used in relevant psychological experiments. The availability of SZU-EmoDage opens up avenues for further research in psychology and related fields, allowing for a deeper understanding of facial perception.

## Background & Summary

Faces contain rich information useful for social interaction^1^. Researchers have widely used facial stimuli to explore cognitive and emotional processing in both healthy individuals and those with disorders^2–4^. Emotional faces are very common stimuli in emotional studies, and standardized emotional face datasets, such as the Radboud Faces Database^5^, FACES^6^, and the American Multiracial Faces Database^7^, have been created to provide research materials with relatively uniform facial features and good image quality. However, researchers found significant cross-cultural differences in emotion recognition among different races^8^.

To overcome the influence of culture on facial expression recognition, researchers in China have established a localized emotion face database based on the basic emotion model (including happiness, anger, fear, sadness, disgust, and surprise). The most widely used database is the Chinese Affective Face Picture System^9^, which was collected from recruited actors, when they imitate different emotions. However, some of the expressions may appear exaggerated or artificial to observers. Furthermore, as some of the volunteers may not present all six emotions well, some expressions are missing for many subjects in the datasets. Therefore, a more natural and standardized Chinese emotional face dataset is needed.

Our faces undergo changes as we grow older. However, age information is rarely considered in face-related studies. Initial research has shown that individuals’ social judgements of young faces primarily consisted of two dimensions: trustworthiness and dominance^10,11^. Nevertheless, with the inclusion of age as a factor and diverse age faces for observers to evaluate, attractiveness emerged as the third dimension in facial social judgments^12^. Moreover, classical face models propose that age, along with emotion and sex, plays a critical role in facial preception^13^. These findings underscore the importance of studying facial age as a critical factor in social judgments. While facial age information is frequently unavailable in current facial datasets, impeding advancements in age-related research on faces.

Additionally, dynamic facial expressions are often seen in social interactions. Dynamic facial expressions evoke stronger emotional responses compared to static ones and are easier to recognize with higher accuracy^14,15^. The dynamic face dataset contributes to explain how people recognize the dynamic properties of faces. While researchers have created a dynamic face dataset based on Caucasian women and men^14^, the Chinese version is missing. It is necessary to establish a Chinese dynamic facial expression dataset, which can effectively capture the characteristics of dynamic facial expression changes in Chinese individuals and provide valuable support for cross-cultural comparisons.

Though several Chinese facial datasets are available^9,16,17^, the authenticity of facial expressions exhibited by volunteers, as well as the diversity among ages of faces, is limited. The credibility and validity of research findings based on such datasets are thus compromised. Furthermore, collecting a large number of volunteers across diverse ages is challenging and requires a substantial investment of time and resources to train these volunteers to exhibit the required emotions on their faces. The adoption of recent AI (Artificial Intelligence) technologies can help overcome this bottleneck in data collection^18^.

Compared to collecting real faces, using AI-generated faces offers advantages in terms of increased experimental control, standardization, and the ease of obtaining novel stimuli^7^. We propose a method that introduces the facial action units into pre-trained StyleGAN to achieve high-quality expression editing. The approach produces naturally synthesized expressions without artifacts. Furthermore, we trained our model using Chinese faces with well-controlled identities, resulting in the generation of consistent basic emotions for each individual. Additionally, our method also includes the functions of age progression and dynamic attribute editing. This proposed method can serve as an extension of the currently available facial datasets, enhancing their quality, authenticity, and diversity.

In this study, the Generative Adversarial Networks (GAN) technique, namely the StyleGAN model, was employed to generate facial images. Our contribution includes the creation of a comprehensive face dataset called SZU-EmoDage, which comprises facial images of 120 individuals (equally divided between men and women) with six basic emotions, various ages, and dynamic emotions. Specifically, the StyleGAN model enables the manipulation of facial expressions and age, to produce all six distinct basic facial emotions for each individual. To meet the growing interest in understanding facial age perception, facial images representing ages ranging from 10 to 70 in 10-year increments were also generated. Notably, the SZU-EmoDage dataset incorporates dynamic and continuous changes in facial expressions, providing a valuable resource for further research in the field.

In summary, we present SZU-EmoDage, the first facial dataset synthesized using AI technologies, for face perception study. Notably, the authenticity of expressions and the diversity of faces across different age groups surpass that of existing face datasets. This dataset makes a valuable contribution to the field of facial perception, particularly in areas such as cross-cultural analysis, dynamic facial perception, and facial age perception. Additionally, the extensive variation in face material can serve as an effective tool for detecting mental disorders. The dataset generated in this study represents a significant expansion of currently available facial materials and is very likely to have a profound impact on related research, owing to its improved quantity and diversity.

## Methods

### Participants

We recruited 120 participants (including 60 men and 60 women, aged from 18 to 28, *M* ± *SD*: 20.47 ± 1.83) to finish the study. All the participants reported no history of mental illness, and had normal or corrected-to-normal vision. All the participants have signed the informed consent form; and we followed the principle of voluntary withdrawal and no harm. After participants finished the experiment, they were paid 100 RMB. The study was performed in agreement with the Declaration of *Helsinki* and approved by the local ethics committee of Shenzhen University.

### Procedure

The procedure can be summarized into three parts: (1) To ensure that the generated faces align with Chinese facial features, we used the open face datasets^5,9,17,19,20^ to train a StyleGAN-based editing model and applied the model to transform a neutral face to six different expressions. All the data we used for the research was obtained with informed consent from the participants. (2) In the process of transforming a neutral face into different expressions, interpolations of latent vectors were employed. This technique enabled the generation of dynamic expressions with varying intensities. (3) Finally, to generate neutral faces of different ages, the open-source SAM (Style-based Age Transform) model^21^ was used. By starting with the neutral face of a subject, this model was able to generate faces ranging from 10 years old to 70 years old.

Specifically, we used StyleGAN^22^ based AU (Action Unit) editing to change the expression of facial images. AU is the contraction or relaxation of one or more muscles of the face. As facial expressions can be decomposed into a combination of multiple AU^23^, the change of a group of AUs can lead to the synthesis of desired expressions on a facial image.

Our model comprises three main modules: the StyleGAN encoder, the AU fusion module, and the StyleGAN generator. The StyleGAN encoder utilizes the encoder architecture and pretrained model from Pixel2Style2Pixel^24^, and remains unchanged throughout training. Its primary function is to extract image features and encode them into the latent space of StyleGAN, to obtain the corresponding latent vector for the image. The AU fusion module consists of the AU encoder, Style extractor, and Style fusioner. The AU encoder maps the input target AU intensity vector to the space of the latent vector, capturing specific attributes of AUs and target expression information. In this mapping process, a 5-layer multi-layer perceptron (MLP) is employed as the AU encoder. Both the Style extractor and Style fusioner also use a 5-layer MLP. The Style extractor extracts features such as identity and background from the latent vector, which are then concatenated with the target AU latent vector obtained from the AU encoder. The resulting concatenated vector is then input to the Style fusioner, which combines style attribute features with expression features, and generates a new latent vector with the desired AU. Through the AU fusion module, manipulation of AU and expression in the latent space can be achieved. The StyleGAN generator utilizes the state-of-the-art StyleGAN ffhq pretrained model^25^, and remains unchanged throughout training, which output the face with desire expression, given the latent vector with the target AU.

**Fig. 1:**
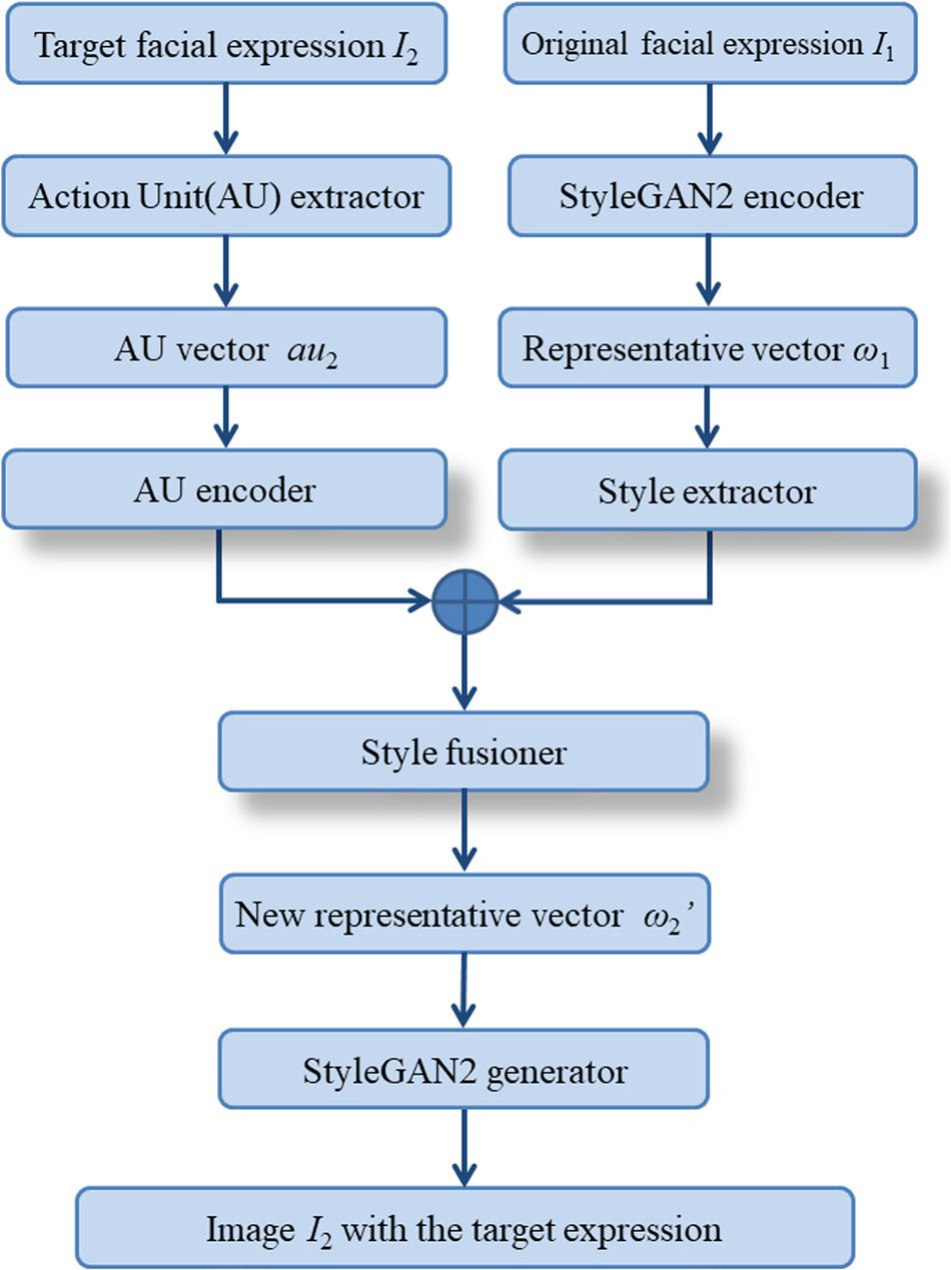
The workflows of editing the facial expression.

In the training process, we paired different expression images of the same person to obtain the original expression image I_1_ and the target expression image I_2._ Then we obtained the latent vector w_1_ corresponding to image I_1_ by StyleGAN encoder^24^, and an AU vector au_2_ representing the contraction or relaxation of 17 AUs of face image I_2_ using AU extractor^26^. The latent vector w_1_ is input into the Style extractor to extract style features, which are then concatenated with the result obtained from the target expression AU vector au_2_ fed into the AU encoder, and then fed into the Style fusioner to obtain a new latent vector w_2_’ for the target expression. Finally, w_2_’ was fed into the StyleGAN generator^22^ to generate the synthesized face image I_2_’ with the target expression. To generate different expressions of a face image I_s_, a set of AU vectors AU_t_ = (au_t1_, …, au_t7_) of 7 target expressions (including neutral) were extracted from the reference images with seven expression labels. The latent vector w_s_ of I_s_ was then input together with the target AU vector au_ti_ (i ∈ [1,7]) into the trained model to obtain the latent vector w_t_, which was then used by StyleGAN generator to synthesize a face image I_t_ with the target expression (Fig. 1).

All images were mapped by StyleGAN into a smooth latent space, W. Two latent vectors with close distances in the latent space will generate similar images. As a result, interpolation in the latent space W can be used to generate intermeddle expressions between the face with original expression I_s_ and the target expression I_t_. Specifically, we performed linear interpolation between the original expression latent vector w_s_ and the target expression latent vector w_t_ to generate multiple intermediate latent vectors. If the intermediate latent vector is closer to w_s_, the expression image generated by StyleGAN generator is more similar to I_s_, and vice versa. In this way, we obtained many faces with intermediate expressions interpolated between two expression images, which were connected together to form a dynamic group.

For age synthesis, SAM^21^ was used to obtain images with desired age I_age_, which can be mapped into a latent vector w_age_ in the latent space of StyleGAN. GAN prior embedded network (GPEN)^27^ was further used to increase the resolution of facial images. Similar to the interpolation of expressions, the dynamic change of age can be realized through interpolations of latent vectors between faces of different ages. Finally, we generated faces of seven basis, aging faces and emotional dynamic faces of 180 individuals (half men and women) in total.

**Fig. 2:**
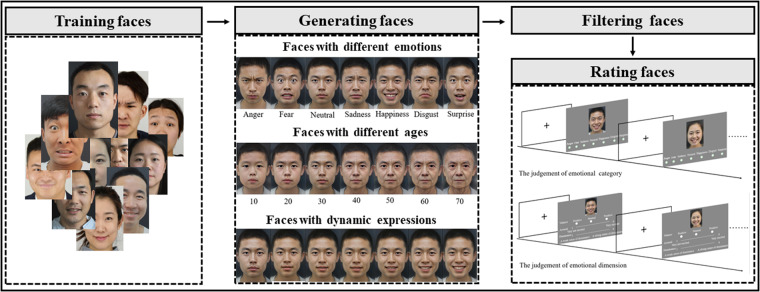
Facial expressions generated by different algorithms.

To validate the efficacy of the proposed data generation method, our method was compared in Fig. 2 to several state-of-the-art expression editing methods including HiSD^28^, GANimation^29^, Expression-manipulator (ExprMAN)^30^, and InterfaceGAN^31^. Each of these methods was utilized to generate neutral and the six basic expressions for the same individual.

**Fig. 3:**
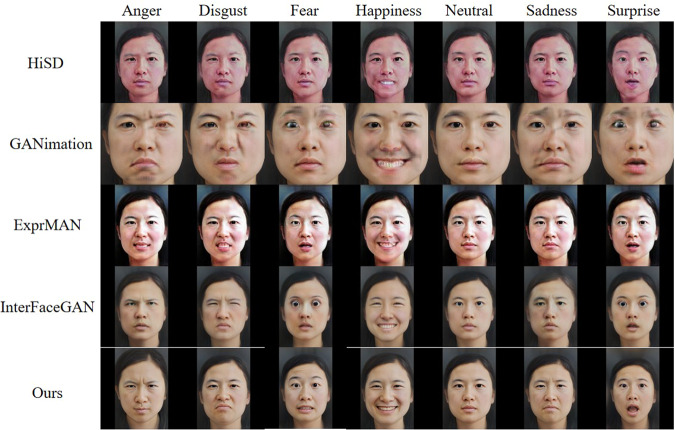
Overview of faces acquisition. The faces dataset includes faces with dynamic expressions, different ages, and emotions.

After using StyleGAN to generate various facial images, we recruited participants to rate the representation of the morphed faces by using the 9-point scale^9^. The development process of this study refers to related facial dateset^9^. Eight participants were firstly invited to evaluate the emotional representation of these pictures and performed a preliminary screening. Finally, the faces of 60 men and 60 women were selected as formal experimental materials. The 120 individuals have 840 emotional faces in total, which is ready to be evaluated for the emotional category and emotional dimension (including valence, arousal and dominance). To prevent fatigue from judging numerous faces, we divided the assessment into 3 parts and recruited 40 college students (20 men and 20 women) in each part. The first group of participants were aged from 18 to 23, (*M* ± *SD*: 19.88 ± 1.65), who were asked to evaluate the emotional category of presented faces. The second group of participants were aged from 18 to 25 (*M* ± *SD*: 20.50 ± 1.88), who were asked to evaluate valence (positive, natural and negative), arousal (from 1 = “very not excited” to 9 = “very excited”), dominance (from 1 = “A weak sense of dominance” to 9 = “A strong sense of dominance”) and the authenticity (from 1 = “not authentic at all” to 9 = “very authentic”) of the faces. The third group of participants were aged from 19 to 28 (*M* ± *SD*: 21.18 ± 1.77), who were asked to evaluate the ages of faces with neutral expression (Fig. 3).

## Data Records

The face dataset is free and available at https://osf.io/7a5fs/ under a CC license^32^. The face images and videos of different emotions, ages and dynamic expressions are stored in three separate compressed folders. Within each folder, different face images or videos generated from the same individualare organized into a subfolder named as “<gender> <id>”, where “gender” and “id” refer to gender and id of the individual. Face images are named according to the corresponding expressions or ages while videos are named according to the corresponding expressions and duration of videos.

## Technical Validation

We conducted a comparative analysis between our method and several state-of-the-art expression editing methods, including HiSD, GANimation, ExprMAN, and InterfaceGAN. Notably, both HiSD and GANimation exhibit limitations in accurately editing the expressions, leading to the generation of low-quality images with noticeable artifacts. Conversely, while InterfaceGAN generates fewer artifacts, it produces expressions that appear unnatural. In comparison, our method excels by producing high-quality images with minimal artifacts and capturing natural expressions, thereby outperforming other methods.

**Fig. 4:**
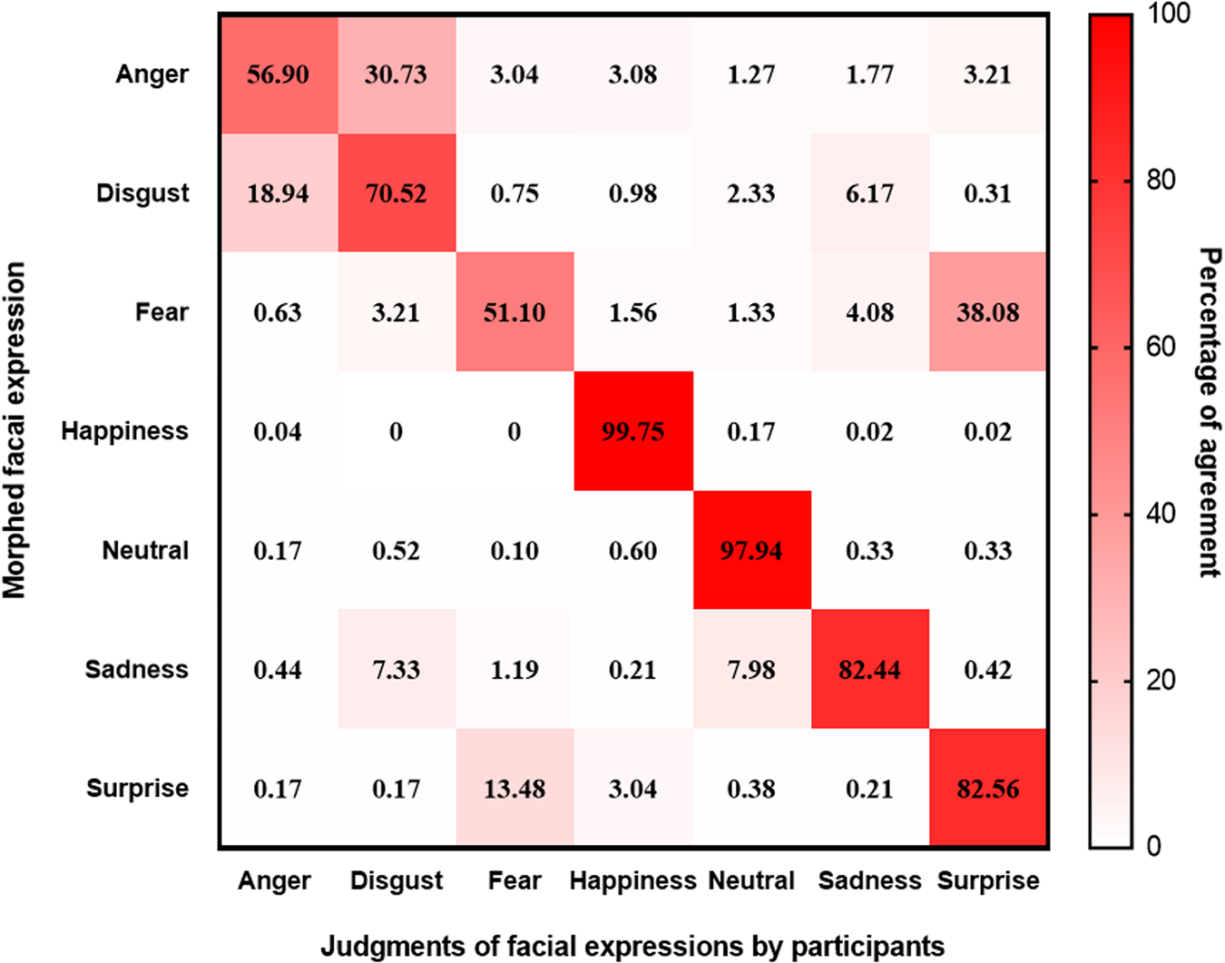
Confusion matrix of rated facial expressions. Columns represent the facial expressions perceived by raters, while rows represent the real expressions.

We compared the expression categories of the 840 faces in our dataset with the categories labeled by volunteers recruited for the study, and the matching proportions are listed in Table 1. On average, the percentages of matching are higher than 70%. Happiness has the highest matching rate (100%), followed by neutral (98%), surprise (83%), sadness (82%), disgust (71%), anger (57%) and fear (51%). Furthermore, a confusion matrix was computed to illustrate the matching rate of each type of facial expressions (Fig. 4).

**Table 1:** The percentage of different matching rates of seven emotions (%).

Rate of acceptance (%)	Anger	Disgust	Fear	Happiness	Neutral	Sadness	Surprise
(0–30)	10.00	1.67	10.83	—	—	0.83	0.83
(30–40)	7.50	2.50	15.83	—	—	—	1.67
(40–50)	16.67	5.00	19.17	—	—	5.00	1.67
(50–60)	11.67	6.67	16.67	—	—	1.67	0.83
(60–70)	22.50	28.33	18.33	—	—	10.00	11.67
(70–80)	20.00	20.83	15.83	—	—	11.67	15
(80–90)	10.83	31.67	2.5	—	2.50	28.33	29.17
(90–100)	0.83	3.33	0.83	100.00	97.50	42.50	39.17

**Fig. 5:**
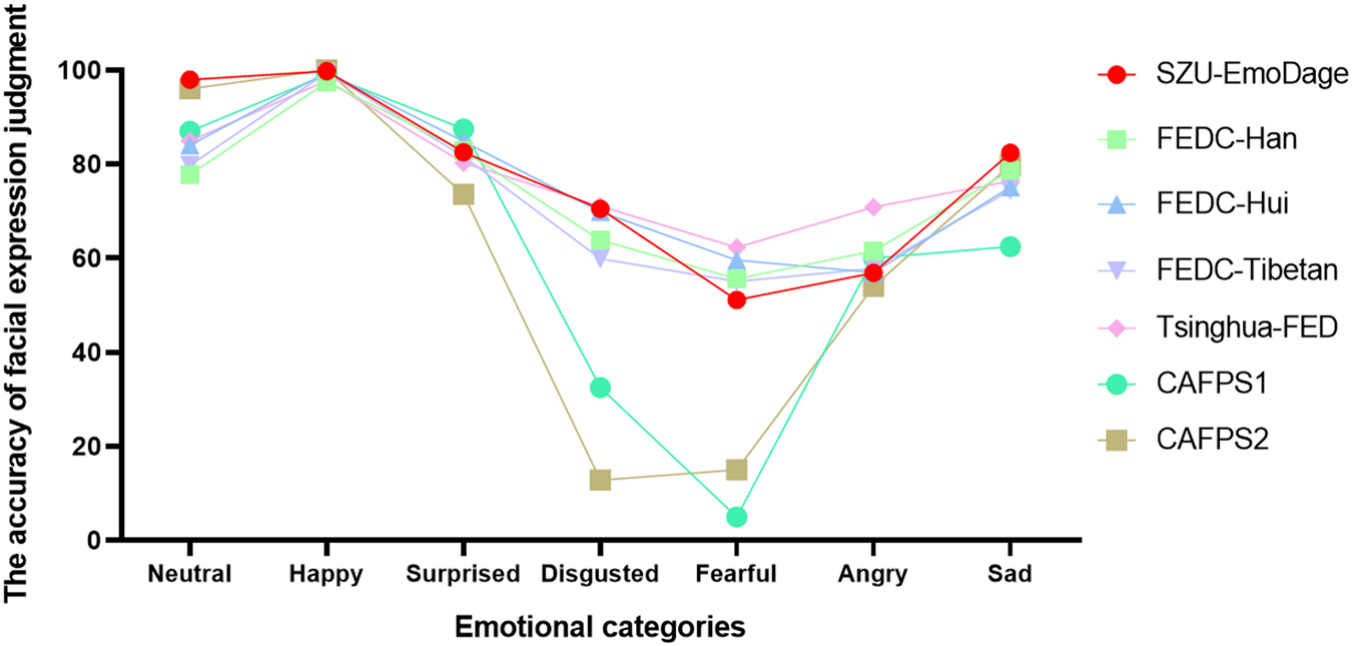
The accuracy rate of basic emotion recognition in SZU-EmoDage, Facial-Expression Database of Chinese Han, Hui, and Tibetan people, Tsinghua facial expression database and two version of Chinese Facial Affective Picture System.

We compared the accuracy of basic emotion recognition in SZU-EmoDage to other Chinese-expression databases, including facial-expression database of Chinese (FEDC)-Han^20^, FEDC-Hui^20^, FEDC-Tibetan^20^, Tsinghua facial-expression database^17^, the first version of CAFPS (CAFPS1)^16^ and the update version of CAFPS (CAFPS2)^33^. The results showed that the accuracy of basic emotion recognition in SZU-EmoDage was similar to that in other databases for neutral, happy, surprised, disgusted, and sad expressions. The accuracy of disgusted and fearful expressions in the two versions of Chinese Facial Affective Picture System was below 30%, while in SZU-EmoDage, it was above 51% (see Table 2 and Fig. 5). The results of this research paper demonstrate the potential of deep learning in emotion recognition and its ability to generate reliable and accurate facial expressions.

**Table 2:** The accuracy rate of basic emotion recognition in different databases (%).

Emotional category	SZU-EmoDage	FEDC-Han	FEDC-Hui	FEDC-Tibetan	Tsinghua-FED	CAFPS1	CAFPS2
Neutral	97.94	77.77	84.06	79.79	84.91	87.00	96.00
Happy	99.75	97.43	99.52	99.48	97.77	99.00	100.00
Surprised	82.56	82.87	84.87	81.67	80.29	87.50	73.60
Disgusted	70.52	63.86	69.85	59.89	71.06	32.50	12.80
Fearful	51.10	55.62	59.61	55.06	62.29	5.00	15.00
Angry	56.90	61.56	56.93	57.81	70.86	60.00	54.00
Sad	82.44	78.63	75.12	74.46	76.41	62.50	79.80

Table 3 shows the percentage of emotional valence rating for each emotion. The results indicated that the majority of negative emotions, including anger, disgust, and sadness, were rated as having a negative emotional valence, with percentages ranging from 65.35% to 68.67%. Fear was also rated as having a negative emotional valence, but with a lower percentage of 37.96%. In contrast, happiness expressions were rated as having a positive emotional valence, with a percentage of 98.08%. Neutral and surprise were rated as having a neutral emotional valence, with percentages of 94.33% and 67.31%, respectively.

**Table 3:** The percentage of the emotional valence rating (%).

Valence	Anger	Disgust	Fear	Happiness	Neutral	Sadness	Surprise
Negative	65.35	68.67	37.96	0.38	2.38	66.77	20.81
Neutral	29.63	28.67	54.67	1.54	94.33	31.60	67.31
Positive	5.02	2.67	7.38	98.08	3.29	1.63	11.88

We compared the arousal and dominance among different emotions. The results showed that happiness was rated as the most arousing emotion, while neutral and disgust were rated as the least arousing. Anger was rated as the most dominant emotion, while a neutral face was rated as the least dominant. To assess the extent to which emotions are expressed naturally, participants were also asked to rate the authenticity of the facial expression. The average authenticity rating for all emotions was above five, indicating that participants perceived the facial expressions as at least somewhat genuine. Pictures of happy expressions were rated as the most authentic (Table 4).

**Table 4:** The degree of arousal, dominance and authenticity of seven emotions.

	Anger	Disgust	Fear	Happiness	Neutral	Sadness	Surprise
Arousal	3.66 ± 1.64	3.51 ± 1.52	4.07 ± 1.69	6.28 ± 1.80	3.51 ± 1.56	3.40 ± 1.45	4.20 ± 1.69
Dominance	4.55 ± 1.90	4.25 ± 1.83	4.20 ± 1.88	4.42 ± 2.36	3.20 ± 1.62	3.90 ± 1.77	3.95 ± 1.78
Authenticity	5.41 ± 1.78	5.28 ± 1.77	5.24 ± 1.84	6.84 ± 1.53	6.10 ± 1.76	5.55 ± 1.70	5.33 ± 1.79

To assess the stability and reliability of facial expressions, we analyzed the internal consistency coefficient of each emotion category in terms of arousal, dominance, and authenticity. The results indicate that all seven emotional categories demonstrated high reliability, suggesting that the evaluation process of selected faces in the database was highly stable and reliable. Cronbach alpha values are all larger than 0.9 (see Table 5).

**Table 5:** The Cronbach alpha internal consistency reliability coefficient of each facial expression in the dimension of arousal, dominance and authenticity.

	Anger	Disgust	Fear	Happiness	Neutral	Sadness	Surprise
Arousal	0.993	0.994	0.994	0.996	0.996	0.994	0.994
Dominance	0.991	0.993	0.993	0.997	0.995	0.993	0.993
Authenticity	0.984	0.987	0.985	0.988	0.993	0.983	0.988

The current dataset also includes faces aged from 10 to 70, with a 10-year interval. The rating results indicate that the proportion of faces in the age ranges of 10–20, 30–50, and 60–70 years old were 25.2%, 34.1%, and 40.7%, respectively.

## Usage Notes

The SZU-EmoDage dataset and the proposed method contribute significantly for face perception related studies. Deep-learning models serve as powerful tools to achieve a trade-off between experimental control and ecological validity^18^, ultimately helps generate naturalistic and standardized datasets. Researchers can leverage our AU-integrated StyleGAN model to generate a large number of faces as required. However, the usage of the method requires some basic technical knowledge, including deep learning fundamentals and proficiency in Python programming, as well as access to computational resources such as GPUs with high memory capacity, to accelerate the image generation process. Additionally, the StyleGAN can be further developed to model new Chinese facial datasets related to social attributes, including facial attractiveness, trustworthiness, and dominance^10–12^. This would allow for the investigation of more scientific questions related to social cognition and the development of new face models for improving facial-perception technology. The generated datasets can also serve as stimuli to detect individual differences in facial expression recognition, particularly those related to emotional disorders, and investigate cross-cultural disparities in facial perception.

## Data Availability

The code we used to generate the morphed faces is publicly available, including Action Unit(AU) extractor (https://github.com/TadasBaltrusaitis/OpenFace), StyleGAN2 encoder (https://github.com/omertov/encoder4editing), StyleGAN2 generator (https://github.com/rosinality/stylegan2-pytorch), SAM (https://github.com/yuval-alaluf/SAM).

## References

[1] Todorov A, Olivola CY, Dotsch R, Mende-Siedlecki P (2015). Social attributions from faces: Determinants, consequences, accuracy, and functional significance. Annu. Rev. Psychol..

[2] Gur RE, Moore TM, Calkins ME, Ruparel K, Gur RC (2017). Face processing measures of social cognition: a dimensional approach to developmental psychopathology. Biol. Psychiatry Cogn. Neurosci. Neuroimaging.

[3] Schindler S, Bublatzky F (2020). Attention and emotion: An integrative review of emotional face processing as a function of attention. Cortex.

[4] Schwartz L, Yovel G (2019). Independent contribution of perceptual experience and social cognition to face recognition. Cognition.

[5] Langner O (2010). Presentation and validation of the Radboud Faces Database. Cogn. Emot..

[6] Ebner NC, Riediger M, Lindenberger U (2010). FACES—A database of facial expressions in young, middle-aged, and older women and men: Development and validation. Behav. Res. Methods.

[7] Chen JM, Norman JB, Nam Y (2021). Broadening the stimulus set: introducing the American multiracial faces database. Behav. Res. Methods.

[8] Mishra MV, Ray SB, Srinivasan N (2018). Cross-cultural emotion recognition and evaluation of Radboud faces database with an Indian sample. PLoS One.

[9] Gong X, Huang YX, Wang Y, Luo YJ (2011). Standardization and Assessment of College Students’ Facial Expression of Emotion. Chin. Ment. Health J..

[10] Oosterhof NN, Todorov A (2008). The functional basis of face evaluation. Proc. Natl. Acad. Sci. USA.

[11] Todorov A, Said CP, Engell AD, Oosterhof NN (2008). Understanding evaluation of faces on social dimensions. Trends Cogn. Sci..

[12] Sutherland CA (2013). Social inferences from faces: Ambient images generate a three-dimensional model. Cognition.

[13] Young AW, Bruce V (2011). Understanding person perception. Br. J. Psychol..

[14] Holland CA, Ebner NC, Lin T, Samanez-Larkin GR (2019). Emotion identification across adulthood using the Dynamic FACES database of emotional expressions in younger, middle aged, and older adults. Cogn. Emot..

[15] Kamachi M (2013). Dynamic properties influence the perception of facial expressions. Perception.

[16] Wang Y, Luo YJ (2005). Standardization and Assessment of College Students’ Facial Expression of Emotion. Chin. J. Clin. Psychol..

[17] Yang T (2020). Tsinghua facial expression database–A database of facial expressions in Chinese young and older women and men: Development and validation. PloS one.

[18] Goetschalckx L, Andonian A, Wagemans J (2021). Generative adversarial networks unlock new methods for cognitive science. Trends Cogn. Sci..

[19] Du S, Tao Y, Martinez AM (2014). Compound facial expressions of emotion. Proc. Natl. Acad. Sci. USA.

[20] Ma J, Yang B, Luo R, Ding X (2020). Development of a facial‐expression database of Chinese Han, Hui and Tibetan people. Int. J. Psychol..

[21] Alaluf Y, Patashnik O, Cohen-Or D (2021). Only a matter of style: Age transformation using a style-based regression model. ACM. Trans. Graph..

[22] Karras T, Laine S, Aila T (2021). A Style-Based Generator Architecture for Generative Adversarial Networks. IEEE Transactions on Pattern Analysis and Machine Intelligence.

[23] Ekman, P. & Friesen, W. V. *The Facial Action Coding System: A Technique for The Measurement of Facial Movement* (Consulting Psychologists Press, San Francisco, 1978)

[24] Richardson, E. *et al*. Encoding in Style: a StyleGAN Encoder for Image-to-Image Translation. in *2021 IEEE/CVF Conference on Computer Vision and Pattern Recognition (CVPR)*, 2287–2296 (2021).

[25] Karras, T. *et al*. Analyzing and Improving the Image Quality of StyleGAN. in *Proceedings of the IEEE/CVF Conference on Computer Vision and Pattern Recognition (CVPR)* (2020).

[26] Baltrusaitis, T., Zadeh, A., Lim, Y. C. & Morency, L.-P. OpenFace 2.0: Facial Behavior Analysis Toolkit. in *2018 13th IEEE International Conference on Automatic Face & Gesture Recognition (FG 2018)* 59–66 (2018).

[27] Yang, T., Ren, P., Xie, X. & Zhang, L. GAN Prior Embedded Network for Blind Face Restoration in the Wild. in *2021 IEEE/CVF Conference on Computer Vision and Pattern Recognition (CVPR)*, 672–681 (2021).

[28] Li, X. *et al*. Image-to-image Translation via Hierarchical Style Disentanglement. in *2021 IEEE/CVF Conference on Computer Vision and Pattern Recognition (CVPR)*, 8635–8644 (2021).

[29] Pumarola, A. *et al.* GANimation: Anatomically-Aware Facial Animation from a Single Image. in *Computer Vision – ECCV 2018* (eds. Ferrari, V., Hebert, M., Sminchisescu, C. & Weiss, Y.), 835–851 (Springer International Publishing, 2018). 10.1007/978-3-030-01249-6_50 PMC6240441 30465044

[30] Ling, J. *et al*. Toward Fine-Grained Facial Expression Manipulation. in *Computer Vision – ECCV 2020* (eds. Vedaldi, A., Bischof, H., Brox, T. & Frahm, J.-M.), 37–53 (Springer International Publishing, 2020).

[31] Shen, Y. *et al*. Interpreting the Latent Space of GANs for Semantic Face Editing. in *2020 IEEE/CVF Conference on Computer Vision and Pattern Recognition (CVPR)*, 9240–9249 (2020).

[32] Han SF (2022). OSF.

[33] Gong X, Huang YX, Wang Y, Luo YJ (2011). Revision of the Chinese Facial Affective Picture System. Chin. J. Clin. Psychol..

